# Clinical and Histopathological Characteristics between Familial and Sporadic Melanoma in Barcelona, Spain

**DOI:** 10.4172/2155-9554.1000231

**Published:** 2014-08-18

**Authors:** Paula Aguilera, Josep Malvehy, Cristina Carrera, Josep Palou, Joan Anton Puig-Butillé, Llúcia Alòs, Celia Badenas, Susana Puig

**Affiliations:** 1 Dermatology Department, Melanoma Unit, Hospital Clínic, IDIBAPS, Barcelona, Spain; 2 CIBER on Rare Diseases, Instituto de Salud Carlos III, Barcelona, Spain; 3 Biochemistry and Molecular Genetics Department, Melanoma Unit, Hospital Clínic, IDIBAPS, Barcelona, Spain; 4 Pathology Department, Melanoma Unit, Hospital Clínic, IDIBAPS, Barcelona, Spain

**Keywords:** Melanoma, Familial melanoma, CDKN2A, Genes, p16, Survivin, Histology, Multiple primary

## Abstract

**Background:**

About 6 to 14% of melanoma cases occur in a familial setting. Germline mutations in CDKN2A are detected in 20 to 40% of melanoma families.

**Objective:**

To characterise the clinical and histopathological characteristics of familial melanoma thus providing more information to clinicians and contribute to the understanding of the genetic-environment interplay in the pathogenesis of melanoma.

**Methods:**

Clinical, histological and immunohistochemical characteristics of 62 familial melanomas were compared with 127 sporadic melanomas.

**Results:**

variables associated with familial melanoma were earlier age at diagnosis (OR 1.036; 95% CI 1.017–1.055), lower Breslow thickness (OR 1.288; 95% CI 1.013–1.683) and in situ melanomas (OR 2.645; 95% CI 1.211–5.778). Variables associated with CDKN2A mutation carriers were earlier age at diagnosis (OR 1.060; 95% CI 1.016–1.105), in situ melanomas (OR 6.961; 95% CI 1.895–25.567), the presence of multiple melanomas (OR 8.920; 95% CI 2.399–33.166) and the immunopositivity of the tumours for cytoplasmic survivin (OR 9.072; 95% CI 1.025–85.010).

**Conclusions:**

Familial melanoma was significantly associated with the earlier age of onset, lower Breslow thickness and with a higher number of in situ melanomas; and also carriers of CDKN2A mutations were associated with a higher risk of multiple melanomas and cytoplasmic survivin immunostaining.

## Introduction

Melanoma (MM) is the human malignancy that has undergone the greatest increase in incidence during the last few decades.

Population Studies suggest that approximately 6 to 14% of melanoma cases occur in a familial setting [1]. In Spain, familial Melanoma is considered when there is at least one invasive melanoma and one more case of melanoma and/or pancreatic cancer among first-degree relatives on the same side of the family [2]. In these families two major melanoma susceptibility genes have been identified. The oncogene *CDK4* has been found in a few melanoma families (estimated 2%) [3]. Germline mutations in *CDKN2A* are found in approximately 20 to 40% of melanoma families [4]. Several studies have reported that patients with melanoma and a *CDKN2A* mutation have an earlier age of onset and an increased risk of multiple primary melanomas (MPM) [5–7]. Not only melanomas occurring in mutation carriers but familial melanoma has been demonstrated to share some characteristics in previous studies [8].

Survivin represents a multifunctional protein that suppresses apoptosis and regulates cell division at the G2-M phase. It is a nuclear shuttle protein that is actively exported from the nucleus [9]. Survivin seems to exist in 2 subcellular pools (in the cytoplasm and nuclear). This is consistent with its function in the regulation of both cell viability and cell division. Growing evidence suggests that survivin expression in cancer cell nuclei may represent an important prognostic marker to predict disease outcome. Current reports in this research area are however inconsistent and propose opposing conclusions regarding the significance and prognostic value of survivin nuclear expression [10,11]. Survivin has been recently identified as a metastasis-associated gene for Melanoma [12].

The purpose of this study was to further characterize and expand the knowledge of the clinical and histopathologic characteristics of familial melanoma to provide more information to clinicians and also contribute to the understanding of the complex interplay of genetic and environmental factors in the pathogenesis of melanoma.

## Material and Methods

The study was approved by the institutional review board.

All familial melanoma patients from whom the paraffin block of the tumour was available were eligible for the study, and two sporadic melanoma patients from whom the paraffin block was also available were also eligible for the study.

We compiled 189 paraffin blocks and the corresponding slides of 189 MMs (62 familial MMs and 127 sporadic MMs). The following variables were evaluated:

### Epidemiological data

Included sex, age, age at diagnosis, histopathological subtype, melanoma site, Breslow thickness, presence of metastases and follow up.

### Phenotype data

Included eye and hair colour, phototype and nevi count.

### Analysis of Histological Features

All histopathological evaluations were carried out on routinely stained HE sections. Cases were classified as superficial spreading melanoma (SSM), lentigo malignant melanoma (LMM), nodular melanoma (NM), acral lentiginous melanoma (ALM) according to the WHO classification [13]. Breslow thickness and Clark level of invasion were evaluated for each tumour. Based on the work of Viros and coworkers [14]. we evaluated the following histological features:

Solar elastosis, type of cells, inflammatory infiltrate, regression, mitotic rate, pagetoid invasion, nest formation, lentiginous hyperplasia and cellular atypia.

### Immunohistochemical analysis TMAs

For immunohistochemical evaluation of all the tumours we constructed tissue microarrays (TMAs). We selected a minimum of 2 areas per tumour and a total of 4 TMAs blocks were performed. Each TMA block was cut into four micrometer sections.

Immunohistochemical studies were performed from tissue microarrays with the automated immunohistochemical system TechMate 500^®^ (Dako Co, Carpinteria, CA), using the EnVision system (Dako). Briefly, 4 µm sections were deparaffinized and hydrated through graded alcohols and water. Peroxidase was blocked for 7.5 minutes in ChemMate peroxidase-blocking solution (Dako). Then, the slides were incubated with the primary antibodies for 30 minutes and washed in ChemMate buffer solution (Dako). The peroxidaselabelled polymer was then applied for 30 minutes. After washing in ChemMate buffer solution, the slides were incubated with the AEC substrate chromogen solution, washed in water, counterstained with hematoxylin and mounted. The primary antibody used in the study was Survivin (Abcam, Cambridge, UK; 1/500 dilution).

### *CDKN2A* mutation analysis

Blood samples were taken from all patients belonging to the familial MM group. The PUREGENE DNA Isolation Kit (Gentra Systems, Minneapolis, MN, USA) was used to isolate genomic DNA from lymphocytes according to the manufacturer’s instructions.

Promoter (−34G>T variant), intronic (IVS2-105) and coding regions of the *CDKN2A* gene (exons 1α, 2 and 3 of the p16INK4A protein and exon 1β corresponding to p14ARF protein) were amplified by PCR using primers and conditions previously described [15].

### Statistical analysis

Descriptive analysis of the sample was performed, including percentages for categorical variables, and mean, minimum, maximum and standard deviation values for continuous variables. Comparisons of continuous variable means were performed using Student’s exact t-test when variables followed a normal distribution. Comparisons of discrete variable means were performed using the Mann–Whitney non parametric test. Comparisons between categorical variables were performed with χ^2^ tests and Fisher corrections were required. Kaplan Meyer analysis and Cox proportional hazards regression models were used to analyze associations between available variables and overall survival. All variables on univariate analysis were incorporated into a multivariable model. Statistical analyses were performed with SPSS (version 18.0; SPSS Inc., Chicago, IL).

## Results

### Descriptive results

We included 189 MMs, 62 (38.8%) belonging to the Familial MM group.

Patients included 88 men (46.6%) and 101 women (53.4%) with a median age of 62.83 year ± 18.35. In the Familial MM group 17 patients (25.8%) were carriers of a *CDKN2A* mutation and 39 (20.6%) of all patients had multiple MM (MMM). For the analysis, the first tumour from 10 MMM patients (25.6%) was included and successive tumours in 29 patients (74.4%). In the familial patients, 37 (61.7%) were the index case and 23 (38.3%) were not. We included two related patients in the familial group.

The median Breslow thickness was 1.53 mm ± 3.072 (min 0.2 max 3mm). Taking into account MM histopathological subtype 128 (67.7%) were SSMM, 14 (7.9%) LMM, 35 (18.5%) ALM and 11 (5.8%) NM.

### Comparison of familiar and sporadic MMs

Of 189 patients included in the study, 62 (38.8%) had a positive family history of melanoma. Patient characteristics and demographic data are presented in Table 1. Patients in the familial melanoma group had a significantly younger age of melanoma onset (median: 44.26 *vs* 56.87), p<0.05. Melanomas belonging to the sporadic melanoma group had a significantly higher Breslow thickness (mean: 2.35 *vs* 1.22), p<0.05. There was a significantly higher number of *in* situ melanomas in the familial group than in the sporadic group (35.5% *vs* 19.7%), p<0.05 (OR. 1.662, 95% CI 1.111–2.485). We found differences, although not significant, in the median Breslow thickness in the MMM group depending on whether the MM was the first or successive (0.64 mm *vs* 0.33 mm), p=0.065. Although not significant, successive MM were more frequently of the *in situ* type than first MM in those patients (48.3% *vs* 20%), p=0.152. We found differences, also not significant, in the median Breslow thickness in the familial group depending on whether the patient was the index case or was not (1.4 mm *vs* 0.73 mm), p=0.713. Non index cases were more frequently of the *in situ* type than index cases (52.2% *vs* 25%), p=0.033 (Table 2). Taking into account melanoma site, a significantly higher percentage of melanomas in the lower limbs was found in the familial melanoma group (30.6% *vs* 13.4%), and no melanoma was found on palms in this group, p<0.05. Comparing the histological subtype of MM between the two groups we found significant differences among SSMM; we found that 82.3% of the familial melanoma tumours were of SSMM type *vs* the 61.4% of the sporadic melanomas, p<0.05.

Phenotypic characteristics and histopathological features of tumours are given in Table 3.

Interestingly a higher number of familial tumours had cytoplasmatic positivity (83.9 *vs* 70.1), p=0.05. Multivariate regression analysis showed that the most representative variables associated with familial melanoma were earlier age at diagnosis (OR 1.036; 95% CI 1.017–1.055), lower Breslow thickness (OR 1.288; 95% CI 1.013–1.683) and *in situ* melanomas (OR 2.645; 95% CI 1.211–5.778) (Table 4).

### Comparison of *CDKN2A* mutated and non mutated MMs

We studied p16 mutations and we found that 17 patients were carriers of a *CDKN2A* mutation, all of them belonging to the familial group. Patient characteristics and demographic data are presented in Table 5. Carriers of a *CDKN2A* mutation had a significantly earlier age of melanoma onset (mean: 37.51 *vs* 54.19 year), p<0.05. Melanomas belonging to non-carriers had a significantly higher Breslow thickness (2.12 *vs* 0.74), p<0.05. There was a significantly higher number of *in situ* melanomas in the mutation carrier group (58.8% *vs* 21.3%), p<0.05. As regards melanoma site, we found a higher number of lower limb melanomas (47.1% *vs* 16.6%), p<0.05. All the tumours in the carrier group were of the SSMM type, p<0.05.

**Figure 1:**
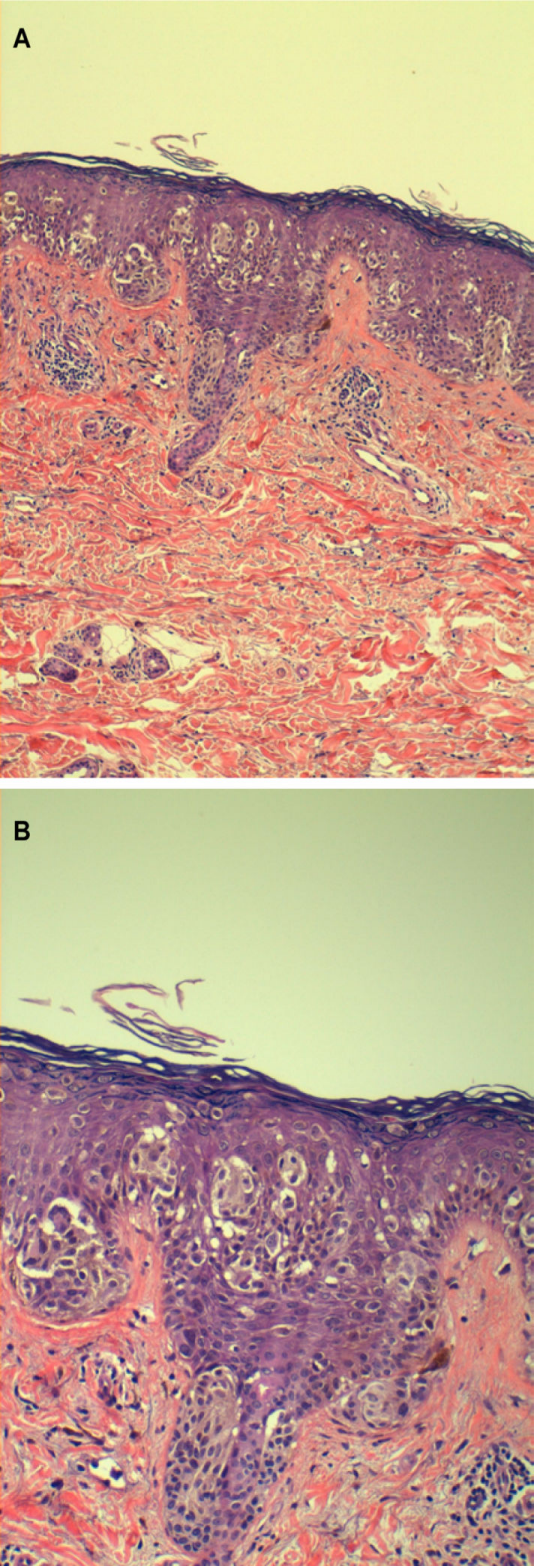
**a and b:** Big round cells in p16 mutated tumour.

Phenotypic characteristics and histopathological features of tumours are given in Table 6. Tumours of *CDKN2A* mutated patients had significantly more big round cells than tumours of non-carriers (35.3% *vs* 4.7%), p<0.05 (Figure 1). No differences were observed in the other histopathological characteristics evaluated. No differences were observed in immuno-positivity for nuclear survivin between the two groups, but a higher number of tumours from *CDKN2A* mutated patients had cytoplasmatic positivity (93.8 *vs* 72.7%), p=0.052.

Multivariate regression analysis showed that the most representative variables associated with *CDKN2A* mutation carriers were earlier age at diagnosis (OR 1.060; 95% CI 1.016–1.105), *in situ* melanomas (OR 6.961; 95% CI 1.895–25.567), the presence of multiple melanomas (OR 8.920; 95% CI 2.399–33.166) and immunopositivity of the tumours for cytoplasmic survivin (OR 9.072; 95% CI 1.025–85.010) (Table 7).

Kaplan Meyer analysis and Cox proportional hazards regression models were used to analyze associations between available variables and overall survival. Mean time of follow up for our patients was 73.78 months (SD 41.53 months, minimum 0 months and maximum 217 months). All the variables in the univariate analysis were included into a multivariate model. On univariate analysis sex, Breslow thickness, Clark level, histopathological subtype, Sporadic or Familial MM group, cellular atypia, inflammatory infiltrate and number of mitoses were associated with overall survival (Table 8). On multivariable analysis, sex carried significant prognostic value for overall survival (hazard ratio (HR) 4.802, 95% confidence interval (CI) 1.779–12.956). Breslow thickness was a significant independent predictor of overall survival (HR:1.571, 95% CI 1.366–1.806). Cellular atypia was also a prognostic value on overall survival on the multivariable model.

## Discussion

We compared the clinical and histopathological characteristics of 62 patients with familial melanoma and 127 patients with sporadic melanoma in our Mediterranean area. We also studied the presence of *CDKN2A* mutation in patients belonging to the familial melanoma group and compared clinical and histopathological characteristics of 17 patients with *CDKN2A* mutation and 168 non-carriers. The characterization of familial cases has been shown to result in early detection of new melanomas by elaborating specific educating and surveillance plans for all the members [16].

Several significant differences were found between familial and sporadic groups. When the variables were included in a multivariable analysis, the earlier age of onset, lower Breslow thickness and a higher proportion of *in situ* melanomas maintained their significance in the model.

Of the 62 familial melanoma patients, 17 were found to be carriers of a *CDKN2A* mutation. Several significant differences were found between carriers and non-carrier groups. When the variables were included in a multivariable analysis the earlier age of onset, a higher proportion of *in situ* melanomas, the higher risk of multiple melanomas and the cytoplasmic survivin immunostaining maintained their significance.

Our results agree with previous reports; other studies reported a younger age at diagnosis of familial melanoma, an increased risk of multiple primary melanomas and a higher proportion of *in situ* and superficial spreading malignant melanoma [6,7]. Nagore and coworker’s also studied a Mediterranean population of familial cases and they found no familial melanoma on hand or foot and no histological ALM. Similarly, in our series we found no familial melanoma on palms and a lower proportion of ALM than in the sporadic group, and no cases of ALM in carriers [6]. An increased proportion of superficial spreading type melanomas in familial melanoma have been reported before [6]. It has been suggested that this is because a relatively large proportion of melanomas in patients with familial melanoma arises from nevi as familial melanoma is associated with increased nevi number. Melanomas that are associated with nevi are usually of the superficial spreading type. Our results are in accordance with this hypothesis as we found a higher proportion of superficial spreading melanomas in the familial and p-16 group and also a larger number of nevi, but we did not analyse the proportion of melanomas arising from nevi.

A higher frequency of melanomas located on the lower limbs was found in familial melanomas and in p-16 melanomas compared with sporadic and non-carriers. In a study by our group Carrera and coworkers found that a higher proportion of the early melanomas on the limbs studied belonged to familial cases [17].

The finding of thinner tumours in familial and p-16 cases could be explained by these patients being high risk patients already under close surveillance, although we and others found that patients with familial melanomas still manifested earlier ages at diagnosis even when only the first cases in each family were considered, which points to an earlier occurrence in this group because of a genetic predisposition [18]. In accordance to that, we found no statistical differences in median Breslow thickness in the familial group depending on whether the patient was the index case or not. The higher frequency of multiple melanomas in individuals with a familial form of cutaneous melanoma is an unquestionable fact, widely reported in the literature and intimately related with the presence of mutations in the *CDKN2A* gene [14]. In our study, the presence of multiple primary melanomas was a predictive factor of being a familial case, and the strongest predictive factor of being a *CDKN2A* carrier, even after multivariate analysis. Our results suggest a higher expression of cytoplasmic survivin in familial and p-16 tumours, a characteristic that is maintained on the multivariate analyses for p-16 tumours. Vetter and co-workers found that cytoplasmic survivin is mainly expressed in metastatic melanomas [12]. We know that somatic loss of p16 is seen in the majority of human melanomas and that this accelerates melanomagenesis [19]. The fact that Vetter and co workers found cytoplasmic survivin mainly expressed in metastatic melanomas could be related to p16 loss, and would be in agreement with our finding that germline p16 mutated melanomas stained for cytoplasmic survivin. The mean Breslow thickness in those p16 germline mutated tumours positive for cytoplasmic survivin was 0.8mm, and we were not able to value survivin as a prognostic marker in this subset of patients because none died or developed metastases.

Our results in the familial and p-16 mutated population are in accordance with the inherited increased susceptibility with, most of them reported before in other founder populations including: (1) younger age of onset; (2) high risk of MPM; (3) lower Breslow thickness and a higher proportion of *in situ* melanomas and (4) higher proportion of cytoplasmic surviving [7].

Taken together, our findings agree with the hypothesis of at least two pathways for cutaneous melanoma pathogenesis proposed by Whiteman and co-worker’s, one associated with increased numbers of nevi, intermittent sun exposure, younger age at diagnosis and location on the trunk (nevus pathway) [20]. The second pathway is associated with chronic sun exposure, fewer nevi, older age at diagnosis, and location in the head and neck region (ultraviolet B pathway). In our familial and p-16 population, the increased number of nevi, the high proportion of superficial spreading type melanomas and low proportion of lentiginous melanomas, the low proportion of tumours located on the head and neck area (with no cases in the p-16 population) and the higher proportion of sunburns during childhood suggest that melanomas in these patients develop predominantly though the nevus pathway.

In summary, our study shows that familial occurrence of cutaneous melanoma was significantly associated with earlier age of onset, lower Breslow thickness and a higher proportion of *in situ* melanomas; and also with higher risk of multiple melanomas and the cytoplasmic survivin immunostaining in the case of carriers of *CDKN2A* mutations. As reported in other founder populations with other *CDKN2A* mutations our findings are in concordance with the so-called divergent pathways hypotheses: familial melanomas tend to follow the nevus pathway.

These findings were based on patients with melanoma in the Mediterranean area. More studies are necessary to determine whether our results apply to other populations.

## Figures and Tables

**Table 1:** Patient and tumour characteristics. Familial versus Sporadic group.

Features	Familial Melanoma (N=62)	Non Familial Melanoma (N=127)	P	CI 95%	OR
Age: Mean (SD)	55.47 (15.980)	66.60 (18.042)	0	6.016–16.255	
Sex: Men	24 (38.7%)	64 (50.4%)	0.131		
Women	38 (61.3%)	63 (49.6%)			
Age at diagnosis			0	7.226–18.004	
Mean (SD)	44.26 (17.198)	56.87 (18.267)			
Melanoma site			0.01		
-head/neck	4 (6.5%)	20 (15.7%)			
-upper limbs	6 (9.7%)	15 (11.8)			
-lower limbs	19 (30.6%)	17 (13.4%)			
-palms	0 (0%)	10 (7.9%)			
-soles	7 (11.3%)	19 (15%)			
-trunk	26 (41.9%)	46 (36.2%)			
Breslow thickness: Mean (SD)	0.79 (1.634)	2.34 (3.79)	0.021	0.169–2.37	
MM in situ	22 (35.5%)	25 (19.7%)	0.018	1.111–2.485	1.662
Histological subtype			0.031		
-LMM	3 (4.8%)	11 (8.7%)			
-SSM	51 (82.3%)	78 (61.4%)			
-NM	1 (1.6%)	10 (7.9%)			
-ALM	7 (11.3%)	28 (22%)			
Multiple Melanomas	20 (32.3%)	19 (15%)	0.006	1.229–2.730	1.832
Past history of sunburns	35 (87.5%)	50 (61.7%)	0.004	1.264–6.951	2.965
Intense solar exposure before 18 years	18 (45%)	18 (21%)	0.006	1.063–2.132	1.506

**Table 2:** Multiple and familial melanoma. Mean Breslow thickness and percentage of in situ type melanomas.

**Multiple Melanoma**	**Mean Breslow**	**p**	***In situ***	**p**
First tumor N=10 (25.6%)	0.64	0.065	20%	0.152
Successive N= 29 (74.4%)	0.33		48.30%	
**Familial Melanoma**	**Mean Breslow**	**p**	***In situ***	**p**
Index case N=37 (61.7%)	1.4	0.713	25%	0.033
Not index case N=23 (38.3%)	0.73		52.20%	

**Table 3:** Phenotypic characteristics and histopathological features. Familial versus Sporadic group.

Features	Familial Melanoma (N=62)	Non Familial Melanoma (N=127)	P
Phototype			0.989
-I–II	22 (44%)	43 (43.9%)	
-III–IV	28 (56%)	55 (56.1%)	
Hair colour			0.138
-brown/black	36 (67.9%)	75 (78.9%)	
-blond/red	17 (32.1%)	20 (21.1%)	
Eye colour			0.441
-dark	31 (58.5%)	61 (64.9%)	
-fair	22 (41.5%)	33 (35.1%)	
Nevi count			0.021
≤ 50	22 (46.8%)	49 (68.1%)	
≥ 50	25 (53.2%)	23 (31.9%)	
Elastosis			0.086
-not present	51 (82.3%)	89 (70.6%)	
-present	11 (17.7%)	37 (29.4%)	
Type of cells			0.663
-epithelioid	45 (72.6%)	101 (79.5%)	
-sarcomatoid	4 (6.5%)	6 (4.7%)	
-big round	7 (11.3%)	7 (5.5%)	
-fusocellular	4 (6.5%)	9 (7.1%)	
-dendritic	2 (3.2%)	4 (3.1%)	
TIL			0.713
-not present	10 (16.1%)	16 (12.6%)	
-mild	45 (72.6%)	99 (78%)	
-severe	7 (11.3%)	12 (9.4%)	
Regression			0.236
-present	13 (21%)	18 (14.2%)	
-not present	49 (79%)	109 (85.8%)	
Mitoses			0.042
-1	46 (74.2%)	75 (59.1%)	
≥ 1	16 (25.8%)	52 (40.9%)	
Pagetoid invasion			0.976
-not present			
-mild	1 (1.6%)	2 (1.6%)	
-severe	20 (32.3%)	39 (30.7%)	
	41 (66.1%)	86 (67.7%)	
Nests			0.517
-present	47 (77%)	103 (81.1%)	
-not present	14 (23%)	24 (18.9%)	
Lentiginoushyperplasia			0.722
-present			
-not present	31 (50%)	60 (47.2%)	
	31 (50%)	67 (52.8%)	
Cellular atypia			0.996
-mild	15 (24.2%)	31 (24.4%)	
-moderate	31 (50%)	64 (50.4%)	
-severe	16 (25.8%)	32 (25.2%)	
Nuclear Survivin			0.821
-positive	13 (23.2%)	29 (24.8%)	
-negative	43 (76.8%)	88 (75.2%)	
Cytoplasmic Survivin			0.05
-positive	47 (83.9%	82 (70.1%)	
-negative	9 (16.1%)	35 (29.9%)	

**Table 4:** Multivariate analysis for characteristics associated with familial melanoma.

Variables	Univariate *			Multivariate **		
	P	OR	95% CI	P	OR	95% CI
Age at diagnosis	0		7.226–18.004	0	1.036	1.017–1.055
Breslow	0.021		0.169–2.025	0.049	1.288	1.013–1.683
MM in situ	0.018	1.662	1.111–2.485	0.015	2.645	1.211–5.778

* Only significant variables in the multivariate logistic regression model are included in the table.

** Forward stepwise multivariate logistic regression.

**Table 5:** Patient and tumour characteristics Carriers of a CDKN2A mutation versus non-carriers.

Features	CDKN2A Melanoma (N=17)	Non CDKN2A Melanoma (N=168)	P	CI 95%	OR
Age: Mean (SD)	49.53 (12.674)	64.29 (17.973)	0	7.782–21.731	
Sex: Men	6 (35.3%)	80 (47.3%)	0.342		
Women	11 (64.7%)	89 (52.7%)			
Age at diagnosis			0	8.567–24.402	
Mean (SD)	37.71 (14.581)	54.19 (18.502)			
Melanoma site			0.015		
-head/neck	0 (0%)	23 (13.6%)			
-upper limbs	1 (5.9%)	19 (11.2%)			
-lower limbs	8 (47.1%)	28 (16.6%)			
-palms	0 (0%)	10 (5.9%)			
-soles	0 (0%)	26 (15.4%)			
-trunk	8 (47.1%)	63 (37.3%)			
Breslow thickness: Mean (SD)	0.74 (0.532)	2.12 (3.490)	0	0.823–1.910	
MM *in situ*	10 (58.8%)	36 (21.3%)	0.002	1.756–10.765	4.348
Histological subtype			0.031		
-LMM	0 (0%)	14 (8.3%)			
-SSM	17 (100%)	109 (64.5%)			
-NM	0 (0%)	11 (6.5%)			
-ALM	0 (0%)	35 (20.7%)			
Multiple Melanomas	11 (64.7%)	28 (16.6%)	0	2.727–17.513	6.91
Past history of sunburns	7 (100%)	76 (67.9%)	0.1		
Intense solar exposure before 18 year	8 (80%)	27 (23.9%)	0.001		

**Table 6:** Phenotypic characteristics and histopathological features Carriers of CDKN2A mutation versus non-carriers

Features	CDKN2A Melanoma (N=17)	Non CDKN2A Melanoma (N=168)	P
**Phototype**			0.919
-I–II	6 (42.9%)	58 (44.3%)	
-III–IV	8 (57.1%)	73 (55.7%)	
**Hair colour**			0.555
-brown/black	11 (68.8%)	97 (75.2%)	
-blond/red	5 (31.3%)	32 (24.8%)	
**Eye colour**			0.833
-dark	9 (60%)	81 (62.8%)	
-fair	6(40%)	48 (37.2%)	
**Nevi count**			0.055
≤ 50	5 (35.7%)	65 (62.5%)	
≥ 50	9 (64.3%)	39 (37.5%)	
**Elastosis**			0.569
-not present	14 (82.4%)	125 (74.4%)	
-present	3 (17.6%)	43 (25.6%)	
**Type of cells**			0
-epithelioid	10 (58.5%)	133 (78.7%)	
-sarcomatoid	1 (5.9%)	9 (5.3%)	
-big round	6 (35.3%)	8 (4.7%)	
-fusocellular	0 (0%)	13 (7.7%)	
-dendritic	0 (0%)	6 (3.6%)	
**TIL**			0.441
-not present	4 (23.5%)	22 (13%)	
-mild	11 (64.7%)	131 (77.5%)	
-severe	2 (11.8%)	16 (9.5%)	
**Regression**			0.742
-present	2 (11.8%)	29 (17.2%)	
-not present	15 (88.2%)	140 (82.8%)	
**Mytoses**			0.242
-1	13 (76.5%)	105 (62.1%)	
≥ 1	4 (23.5%)	64 (37.9%)	
**Pagetoid invasion**			0.34
-not present	1 (5.9%)	2 (1.2%)	
-mild	5 (29.4%)	54 (32%)	
-severe	11 (64.7%)	113 (66.9%)	
**Nests**			0.743
-present	14 (87.5%)	134 (79.3%)	
-not present	2 (12.5%)	35 (20.7%)	
**Lentiginous hyperplasia**			0.945
-present			
-not present	8 (47.1%)	81 (47.9%)	
	9 (52.9%)	88 (52.1%)	
**Cellular atypia**			0.408
-mild	2 (11.8%)	42 (24.9%)	
-moderate	9 (52.9%)	85 (50.3%)	
-severe	6 (35.3%)	42 (24.9%)	
**Nuclear Survivin**			1
-positive	4 (25%)	38 (24.7%)	
-negative	12 (75%)	116 (75.3%)	
**Cytoplasmic Survivin**			0.052
-positive	15 (93.8%)	112 (72.7%)	
-negative	1 (6.3%)	42 (27.3%)	

**Table 7:** Multivariate analysis for characteristics associated with p-16 melanoma.

Variables	Univariate*			Multivariate**		
	P	OR	95% CI	P	OR	95% CI
Age at diagnosis	0		8.567–24.402	0.007	1.06	1.016–1.105
MM *in situ*	0.002	4.348	1.756–10.765	0.003	6.961	1.895–25.567
Multiple MM	0	6.91	2.727–17.513	0.001	8.92	2.399–33.166
Cytoplasmatic survivin +	0.052	0.197	0.027–1.447	0.049	9.072	1.025–85.010

* Only significant variables in the multivariate logistic regression model are included in the table.

** Forward stepwise multivariate logistic regression.

**Table 8:** Kaplan Meyer analysis and Cox proportional hazards regression models results.

Variable	Survival days	Univariate analysis		Multivariable analysis		
		Log Rank	P Value	Hazard Ratio	95% CI	P Value
**Sex**				4.802	1.779–12.956	0.002
-Men	5279.871	3.922	0.048			
-Women	5713.971					
**Breslow**				1.571	1.366–1.806	0
≤ 1 mm	6348.898	28.948	0			
≥ 1 mm	2996.119					
**Breslow**						
≤ 2 mm	6108.325	52.918	0			
≥ 2 mm	2120.635					
**Breslow**						
≤ 5 mm	5948.213	110.925	0			
≥ 5 mm	917.667					
**Clark level**						
-I	6470.2	61.614	0			
-II	5985.921					
-III	4631.688					
-IV	1944.356					
-V	1285.625					
**Tumor Location**		6.079	0.299			
**Group**						
-Sporadic MM	5264.739	7.456	0.006			
-Familiar MM						
	5995.199					
**MMM**						
-Yes	6072.296	1.505	0.22			
-No	4236.897					
**Nuclear Survivin**						
-Positive		0.499	0.48			
-Negative	4555.304					
	4622.521					
**Cytoplasmic**						
Survivin		0.033	0.856			
-Positive	4643.208					
-Negative	4369.296					
**Cellular atypia**				2.386	2.137–5.869	0.049
-1	6212.268	13.461	0.001			
-2	4582.507					
-3	4498.736					
**Inflammatory infiltrate**						
0		8.077	0.044			
-1						
-2						
-3						
**Mytoses**						
≤ 1	6208.659	20.441	0			
≥ 1	4229.195					
**Pagetoid invasion**		2.049	0.562			
Nests		1.975	0.16			
Elastosys		1.582	0.663			
